# Differential roles of the hemerythrin-like proteins of *Mycobacterium smegmatis* in hydrogen peroxide and erythromycin susceptibility

**DOI:** 10.1038/srep16130

**Published:** 2015-11-26

**Authors:** Xiaojing Li, Jingjing Li, Xinling Hu, Lige Huang, Jing Xiao, John Chan, Kaixia Mi

**Affiliations:** 1 CAS Key Laboratory of Pathogenic Microbiology and Immunology, Institute of Microbiology, CAS, Beijing 100101, China; 2 School of Life Sciences, Anhui University, Hefei 230601, Anhui Province, China; 3 Division of Infectious and Diseases, Department of Medicine, Albert Einstein College of Medicine, New York 10461, USA; 4 Beijing Key Laboratory of Microbial Drug Resistance and Resistome, Beijing 100101, China

## Abstract

Hemerythrin-like proteins are oxygen-carrying non-heme di-iron binding proteins and their functions have effect on oxidation-reduction regulation and antibiotic resistance. Recent studies using bioinformatic analyses suggest that multiple hemerythrin-like protein coding sequences might have been acquired by lateral gene transfer and the number of hemerythrin-like proteins varies amongst different species. *Mycobacterium smegmatis* contains three hemerythrin-like proteins, MSMEG_3312, MSMEG_2415 and MSMEG_6212. In this study, we have systematically analyzed all three hemerythrin-like proteins in *M. smegmatis* and our results identified and characterized two functional classes: MSMEG_2415 plays an important role in H_2_O_2_ susceptibility, and MSMEG_3312 and MSMEG_6212 are associated with erythromycin susceptibility. Phylogenetic analysis indicated that these three proteins have different evolutionary origins, possibly explaining their different physiological functions. Here, combined with biological and phylogenetic analyses, our results provide new insights into the evolutionary divergence of the hemerythrin-like proteins in *M. smegmatis.*

Hemerythrin-like proteins are non-heme, di-iron and O_2_-binding proteins that are ubiquitous from bacteria to mammals and function in oxygen storage and transport. Bioinformatic evidence indicates that prokaryotic genomes collectively encode hundreds of hemerythrin-like proteins^1^,^2^,^3^. Based on the structural characterization of hemerythrin-like proteins, their functions are correlated with redox regulation in bacteria. One study showed that the hemerythrin-like protein in *Methylococcus capsulatus* functions as an oxygen-carrier^4^, while the hemerythrin-like protein in *Campylobacter jejuni* acts to protect iron-sulfur cluster enzymes from oxidative damage^5^. Although hundreds of hemerythrin-like proteins have been predicted in bacteria, studies on the biological functions of hemerythrin-like proteins are few. One study showed that the multi domain protein VcBhr-DGC (with a hemerythrin domain and a diguanylate cyclase GGDEF domain) functions as a regulatory oxygen sensor for switching between reducing or anaerobic environments in *Vibrio cholerae*^6^. This is the first demonstration of a regulatory function for a hemerythrin domain and hints that hemerythrin-like proteins might have other unannotated functions.

The number of hemerythrin-like proteins differs from strain to strain and this variation is predicted to be related to differences in the oxygen concentration of the environment^2^. A number of hemerythrin-like proteins have been found in magnetotactic bacteria and their functions are predicted to be correlated with bacterial physiological conditions (survival under certain oxygen tensions or high concentrations of iron *in vivo*)^2^. However, the functions predicted for many hemerythrin-like proteins are simply based on their molecular sequences. The functions of multiple hemerythrin-like proteins in one organism have not previously been identified. Multiple homologs are common in bacteria and understanding the functional divergence of paralogs in one organism is a challenge in biology, as the multiplicity of genes for hemerythrin-like proteins is an obstacle to study their distinctive individual functions.

The genus *Mycobacterium* is comprised of a number of Gram-positive bacteria, including both pathogens, such as *Mycobacterium tuberculosi*s and *Mycobacterium leprae*, and nonpathogens, such as the soil microorganism *Mycobacterium smegmatis*, which is commonly used in laboratory experiments as a model organism for *M. tuberculosis*^7^. *Mycobacterium* is capable to survive under environmental stresses, such as oxidative stress, hypoxia and exposure to multiple antimicrobial agents^8^,^9^. The identification of undefined proteins and pathways involved into oxidative stress and antimicrobial response might give new insights to understanding the pathogenesis of *M. tuberculosis* and response to antibiotic exposure in mycobacteria^10^,^11^. Mycobacteria are predicted to contain many hemerythrin-like proteins. For example, *M. tuberculosis* (NC_000962.3) has been predicted to contain three hemerythrin-like proteins. Five genes have been predicted to encode hemerythrin-like proteins in *Mycobacterium avium* (NC_008595). *M. smegmatis* possesses three hemerythrin-like proteins, MSMEG_3312, MSMEG_2415 and MSMEG_6212. In this study, we sequentially overexpressed and deleted each of the three genes encoding hemerythrin-like proteins in *M. smegmatis.* We showed that MSMEG_6212 and MSMEG_3312 modulated erythromycin susceptibility and that the resistance of *msmeg_3312* and the *msmeg_6212* double-knockout strain, mc^2^155:Δ*3312-6212,* was similar to single-knockout strains mc^2^155:Δ*3312* and mc^2^155:Δ*6212*. MSMEG_2415 plays a major role in H_2_O_2_ susceptibility but not in erythromycin susceptibility. MSMEG_3312 exhibited only a mild H_2_O_2_ response in mc^2^155:Δ*2415.*

In addition, MSMEG_6212 was not associated with H_2_O_2_ susceptibility; overexpression of *msmeg_6212* in both mc^2^155:Δ*2415* and the mc^2^155:Δ*2415-3312* double-knockout strain did not influence H_2_O_2_ susceptibility relative to the corresponding parental strains. Phylogenetic analysis of bacterial hemerythrin-like proteins showed that three mycobacterial hemerythrin-like proteins are likely derived from different lineages, possibly explaining their different biological functions. Here, combined with analyses of biological function and phylogenetic analyses our results provide new insights into the evolutionary divergence of the hemerythrin-like proteins in *M. smegmatis.*

## Results

### MSMEG_6212 is associated with erythromycin susceptibility

**Figure 1:**
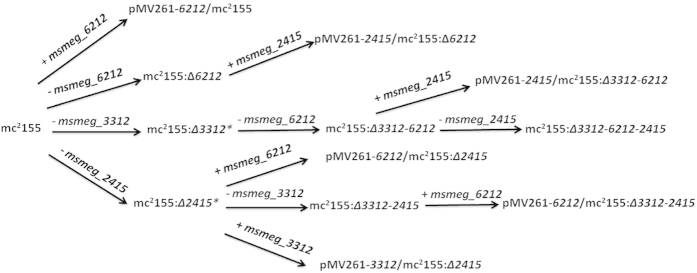
Sequential deletion of hemerythrin-like proteins in *M. smegmatis*. “−” represents in-frame deletions using specialized transduction. “+” represents the overexpression of the indicated genes encoding hemerythrin-like proteins. *Knockout mutants were constructed in previous studies^12^,^13^.

**Figure 2:**
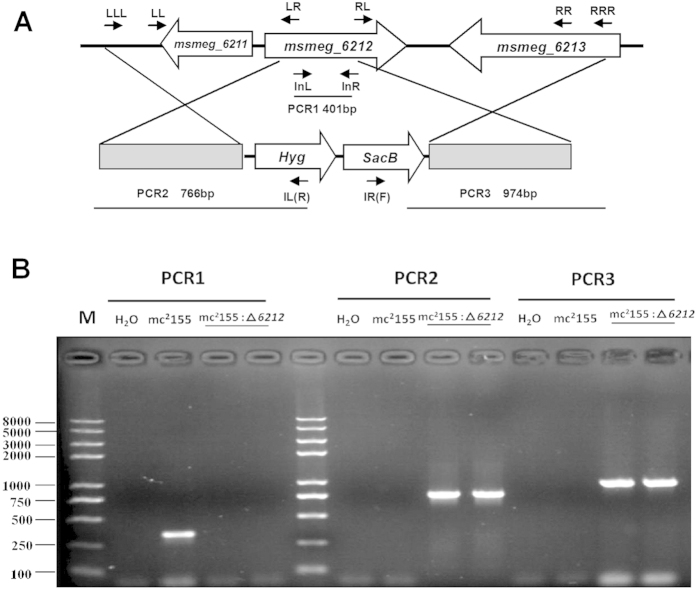
Construction of the mc^2^155:Δ*6212 M. smegmatis* strain. **(A)** The upper panel shows the genetic organization of the *msmeg_6212* gene locus. Gene location and orientation are indicated by large arrows. Primer location and orientation are shown by small arrows. (**B**) The lower panel shows the PCR for verification of the mc^2^155:Δ*6212 M. smegmatis* strain.

**Figure 3:**
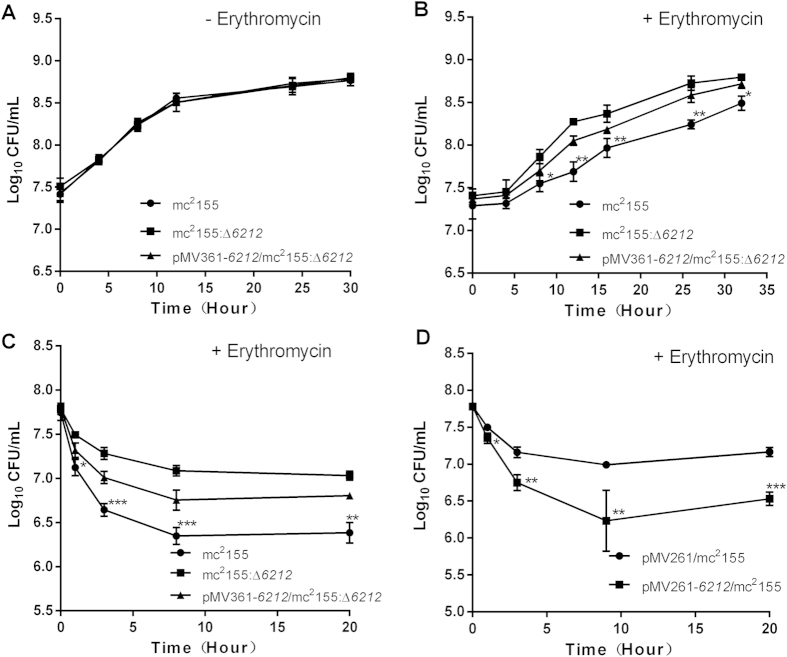
MSMEG_6212 is associated with erythromycin susceptibility. (**A**) Growth curve of mc^2^155, mc^2^155:Δ*6212* and the complemented strain pMV361-*6212*/mc^2^155:Δ*6212* in 7H9 medium. (**B**) Growth curve of mc^2^155, mc^2^155:Δ*6212* and the complemented strain pMV361-*6212*/mc^2^155:Δ*6212* in 7H9 medium with 1.56 mg/L erythromycin (*p < 0.05, **p < 0.01). (**C**) Killing curve for mc^2^155, mc^2^155:Δ*6212* and the complemented strain pMV361-*6212*/mc^2^155:Δ*6212* in 7H9 medium with 31.25 mg/L erythromycin (*p < 0.05, **p < 0.01, ***p < 0.001). (**D**) Killing curve for pMV261/mc^2^155 and pMV261-*6212*/mc^2^155 in 7H9 medium with 15.6 mg/L erythromycin (*p < 0.05, **p < 0.01, ***p < 0.001).

To investigate whether the three *M. smegmatis* hemerythrin-like proteins, MSMEG_3312, MSMEG_2415 and MSMEG_6212, have distinct or overlapping functions, we used a series of strains overexpression individual genes and knockout mutants. The specialized transduction strategy for the sequential deletion of the three genes encoding hemerythrin-like proteins in *M. smegmatis*, and the overexpression of individual genes encoding hemerythrin-like proteins is shown in Fig. 1. We have previously shown that MSMEG_2415 is involved in the SigF-mediated H_2_O_2_ pathway and that MSMEG_3312 is associated with erythromycin susceptibility^12^,^13^. Here, to characterize the function of MSMEG_6212, we constructed a *msmeg_6212* knockout strain (mc^2^155:Δ*6212*). Knockout mutants were confirmed by PCR analysis (Fig. 2B). A *msmeg_6212* gene fragment was not amplified and no *msmeg_6212* mRNA was detected in an assay of its mRNA expression (data not shown). The *msmeg_6212* mutant, mc^2^155:Δ*6212,* was complemented with a single integrated copy using pMV361-*6212.* The constructed *M. smegmatis* mutant strain mc^2^155:Δ*6212* was initially tested for growth in rich 7H9 medium and defined Sauton medium. Growth of mc^2^155:Δ*6212* appeared to have no discernable phenotypic difference from the wild type strain mc^2^155, in either rich (Fig. 3A) or defined media (data not shown). These results indicate that *msmeg_6212,* like the previous investigated *msmeg_3312* and *msmeg_2415,* is not an essential gene for *M. smegmatis* growth in either 7H9 rich medium or Sauton defined medium. In order to characterize the potential roles of MSMEG_6212, we compared the minimum inhibitory concentrations (MICs) of eleven antibiotic drugs, and H_2_O_2_ in the *msmeg_6212* knockout strain mc^2^155:Δ*6212* and wild type strain mc^2^155 (Table S1). Surprisingly, a difference in MIC values was detected only for the macrolides erythromycin and azithromycin (AZM) (Table S1). To clarify the effect of MSMEG_6212 on erythromycin susceptibility, we performed drug exposure experiments to compare the growth rates of wild-type strain mc^2^155, *msmeg_6212* knockout strain mc^2^155:Δ*6212*, and the complemented strain pMV361-*6212*/mc^2^155:Δ*6212* in the presence of 1.56mg/L erythromycin (Fig. 3B). The strain mc^2^155:Δ*6212* showed a growth advantage compared with wild type mc^2^155, which was partially reversed in the complemented strain pMV361-*6212*/ mc^2^155:Δ*6212* in the presence of erythromycin (Fig. 3B). Furthermore, we compared the survival of various *M. smegmatis* strains every few hours under treatment with 31.2 mg/L (10x MIC) erythromycin. As shown in Fig. 3C and Fig. S1, the percentage survival of mc^2^155:Δ*6212* was greater than that of wild type mc^2^155, whereas the complemented strain pMV361-6212/mc^2^155:Δ*6212* did not grow well and its survival was partially reversed to that of the wild-type. As overexpression of MSMEG_6212 increased susceptibility to erythromycin, we used 15.6 mg/L (5 × MIC) to perform the killing experiment: overexpression of *msmeg_6212* caused greater susceptibility to erythromycin and lower survival than in wild type mc^2^155 under the same treatment (Fig. 3D and Fig. S1). Taken together, these results show that, like MSMEG_3312, MSMEG_6212 negatively impacts erythromycin resistance.

### Both MSMEG_3312 and MSMEG_6212 affect MtrA-mediated erythromycin susceptibility and MSMEG_3312, but not MSMEG_6212, has a redundant role in the H_2_O_2_ response

**Figure 4:**
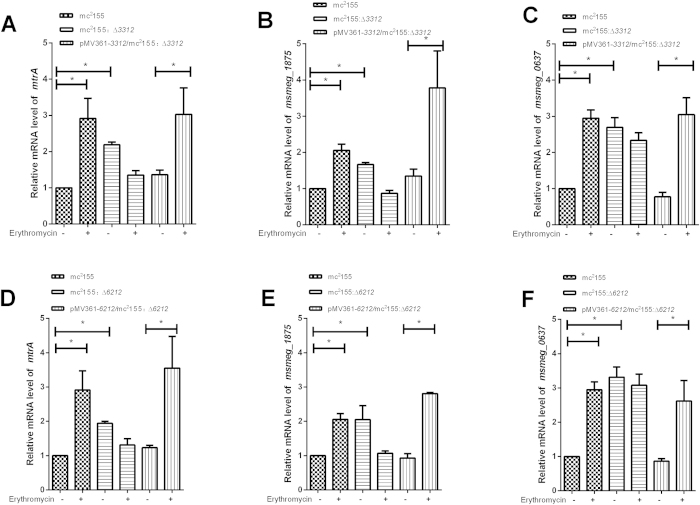
Both MSMEG_3312 and MSMEG_6212 affect MtrA-mediated erythromycin susceptibility. (**A–C**) Relative expression levels of *mtrA* and MtrA*-*regulon genes (*msmeg_1875* and *msmeg_0637*) in mc^2^155, mc^2^155:Δ*3312* and complemented strain pMV361-*3312*/ mc^2^155:Δ*3312* with (**+**) or without (**−**) erythromycin treatment. (**D–F**) Relative expression levels of *mtrA* and MtrA*-*regulon genes (*msmeg_1875* and *msmeg_0637*) in mc^2^155, mc^2^155:Δ*6212* and complemented strain pMV361-*6212*/ mc^2^155:Δ*6212* with (**+**) or without (**−**) erythromycin treatment. Levels of mRNA expression were determined by qRT-PCR. Results are shown as the mean ± standard deviation of three replicates (*p < 0.05). The same wild type was used for evaluation the relative expression levels of *mtrA* and its regulon genes.

Both mc^2^155:Δ*3312* and mc^2^155:Δ*6212* mutant cells were found to be slightly resistant than the wild type strain mc^2^155 under erythromycin treatment (Table S1). When we compared the mRNA levels of transcriptional regulators MtrA and WhiB7, known to affect erythromycin susceptibility^14^,^15^,^16^, among mc^2^155, mc^2^155:Δ*6212* and mc^2^155:Δ*3312*, we found no difference in the level of WhiB7 mRNA between the mc^2^155, mc^2^155:Δ*6212* and mc^2^155:Δ*3312* strains with or without erythromycin treatment (data not shown). This result suggests that WhiB7 responds to erythromycin independent of MSMEG_3312, and MSMEG_6212. In contrast, knockout of *msmeg_3312* led to a 2.19 ± 0.07 fold increase in the mRNA level of *mtrA* relative to wild type mc^2^155, and a 1.94 ± 0.05 fold increase in *mtrA* mRNA in mc^2^155:Δ*6212* (Fig. 4A,D). These increases were partially reversed in the complemented strains pMV361-*3312*/mc^2^155:Δ*3312* and pMV361-*6212*/mc^2^155:Δ*6212* (Fig. 4A,D). These results suggest that both *msmeg_3312* and *msmeg_6212* affect the mRNA level of *mtrA*. We then examined the influence of MSMEG_3312/MSMEG_6212 on the MtrA-mediated erythromycin response pathway. We compared the mRNA level of *mtrA* between mc^2^155, mc^2^155:Δ*6212* and mc^2^155:Δ*3312* after treatment with 3.125 mg/L erythromycin for 30 min. As shown in Fig. 4A,D, *mtrA* mRNA was induced by erythromycin in mc^2^155, while induction of *mtrA* mRNA by erythromycin was not detected in either mc^2^155:Δ*6212* or mc^2^155:Δ*3312*. Induction of *mtrA* mRNA was restored in the complemented strains pMV361-*3312*/mc^2^155:Δ*3312* and pMV361-*6212*/mc^2^155:Δ*6212*. These results show that MSMEG_6212 and MSMEG_3312 are required for the *mtrA*-mediated response to erythromycin.

**Figure 5:**
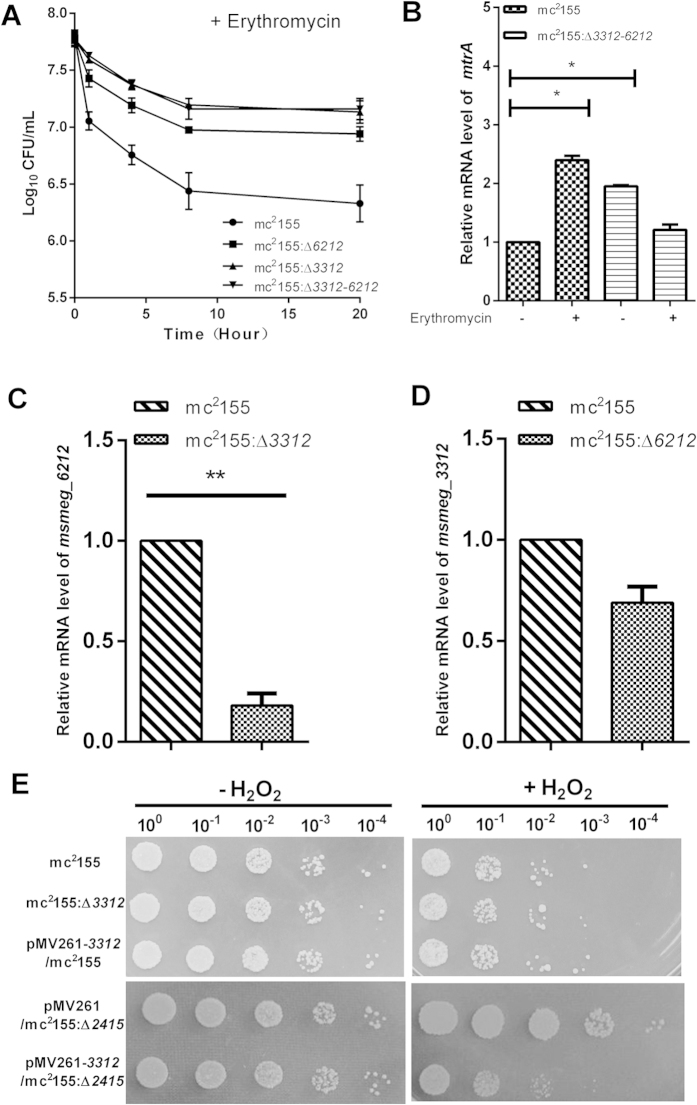
MSMEG_3312 and MSMEG_6212 have redundant roles in erythromycin susceptibility; MSMEG_3312, but not MSMEG_6212, has a redundant role in H_2_O_2_ susceptibility. (**A**) Killing curve for mc^2^155, mc^2^155:Δ*3312,* mc^2^155:Δ*6212* and mc^2^155:Δ*3312-6212* in 7H9 medium with 31.25 mg/L erythromycin. (**B**) Relative expression levels of *mtrA* in mc^2^155 and mc^2^155:Δ*3312-6212* with (**+**) or without (**−**) erythromycin treatment. Levels of mRNA expression were determined by qRT-PCR. Results are shown as the mean ± standard deviations of three replicates (*p < 0.05). (**C**) Relative expression levels of *msmeg_6212* in mc^2^155:Δ*3312*. Levels of mRNA expression were determined by qRT-PCR. Results are shown as the mean ± standard deviation of three replicates (**p < 0.01). (**D**) Relative expression levels of *msmeg_3312* in mc^2^155:Δ*6212*. Levels of mRNA expression were determined by qRT-PCR. Results are shown as the mean ± standard deviation of three replicates. (**E**) H_2_O_2_ resistance phenotype of mc^2^155, mc^2^155:Δ*3312*, pMV261-*3312*/mc^2^155, pMV261/mc^2^155:Δ*2415*, pMV261-*3312*/mc^2^155:Δ*2415*. The pictures shown are representative of three independent experiments.

**Figure 6:**
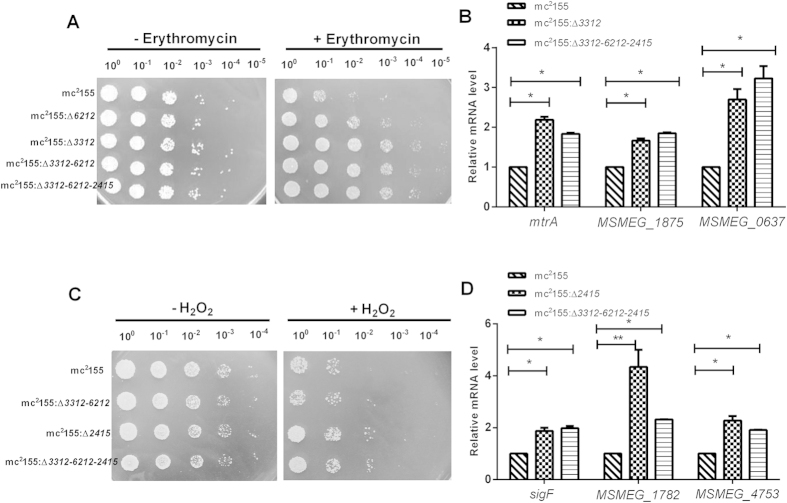
Survival of triple hemerythrin-like gene knockout mc^2^155:Δ*3312-6212-2415* after challenge with H_2_O_2_ and erythromycin. (**A**) Erythromycin resistance phenotype of mc^2^155, mc^2^155:Δ*3312,* mc^2^155:Δ*6212*, mc^2^155:Δ*3312*-*6212* and mc^2^155:Δ*3312-6212-2415*. The pictures shown are representative of three independent experiments. (**B**) Relative expression levels of *mtrA, msmeg_1874*, and *msmeg_0637* in mc^2^155:Δ*3312* and mc^2^155:Δ*3312-6212-2415*. mRNA expression levels were determined by qRT-PCR. Results are shown as the mean ± standard deviation of three replicates (*p < 0.05). (**C**) H_2_O_2_ resistance phenotype of mc^2^155, mc^2^155:Δ*2415*, mc^2^155:Δ*3312-6212* and mc^2^155:Δ*3312-6212-2415*. The pictures shown are representative of three independent experiments. (**D**) Relative expression levels of *sigF, msmeg_1782*, and *msmeg_4753* in mc^2^155:Δ*2415* and mc^2^155:Δ*3312-6212-2415.* mRNA expression levels were determined by qRT-PCR. Results are shown as the mean ± standard deviation of three replicates (*p < 0.05, **p < 0.01).

In addition, we measured changes in the mRNA levels of MtrA regulon genes *msmeg_1875* (encoding sensor histidine kinase MtrB) and *msmeg_0637* (encoding iron-sulfur binding oxidoreductase)^17^ in response to erythromycin treatment in mc^2^155, mc^2^155:Δ*6212*, mc^2^155:Δ*3312* and complemented strains pMV361-*3312*/mc^2^155:Δ*3312* and pMV361-*6212*/mc^2^155:Δ*6212.* The level of *msmeg_1875* and *msmeg_0637* mRNA increased in mc^2^155 in response to erythromycin, but induction of *msmeg_1875* and *msmeg_0637* was abrogated in both mc^2^155:Δ*6212* and mc^2^155:Δ*3312* in response to erythromycin (Fig. 4B,C,E,F). Correspondingly, induction of *msmeg_1875* and *msmeg_0637* was restored in both the complemented strain pMV361-*3312*/ mc^2^155:Δ*3312* and pMV361-*6212*/mc^2^155:Δ*6212* in response to erythromycin (Fig. 4B,C,E,F). Taken together, those results indicate that both MSMEG_6212 and MSMEG_3312 are required for the MtrA-mediated erythromycin response. To characterize the relationship between MSMEG_3312 and MSMEG_6212, we constructed a double knockout mutant strain mc^2^155:Δ*3312-6212* and assayed its resistance to erythromycin. As shown in Figs 5A and 6A, the resistance of the double-knockout mutant mc^2^155:Δ*3312-6212* to erythromycin appeared to be comparable to mc^2^155:Δ*3312.* Moreover, a significant increase of *mtrA* mRNA in mc^2^155 was observed in response to erythromycin (Fig. 5B), while no significant changes of mRNA level in mc^2^155:Δ*3312-6212* was observed in response to erythromycin (Fig. 5B)

The unchanged level of erythromycin resistance in the double-knockout mutant strain mc^2^155:Δ*3312-6212* suggested that there was no cumulative effect in the double-knockout mutant mc^2^155:Δ*3312-6212*. Our results thus indicate that MSMEG_3312 and MSMEG_6212 fall in the same pathway. To determine the order of MSMEG_3312 and MSMEG_6212, we analyzed the level of *msmeg_6212* mRNA expression in mc^2^155:Δ*3312* and of *msmeg_3312* expression in mc^2^155:Δ*6212.* As shown in Fig. 5C, the level of *msmeg_6212* mRNA decreased 4.86 ± 0.70 fold in mc^2^155:Δ*3312* compared with the wild type strain mc^2^155, while the level of *msmeg_3312* mRNA was not significantly different in the *msmeg_6212* knockout strain mc^2^155:Δ*6212* (Fig. 5D). We thus reason that MSMEG_3312 is upstream of MSMEG_6212. Knockout of *msmeg_3312* decreased the level of *msmeg_6212* mRNA and thus dysregulated *mtrA* mRNA.

As the remaining *M. smegmatis* hemerythrin-like protein MSMEG_2415 is involved in the H_2_O_2_ stress response^13^, we also examined the effects of MSMEG_3312 and MSMEG_6212 on H_2_O_2_ susceptibility. Cells of strain mc^2^155:Δ*3312* were treated with 5 mM H_2_O_2_ for 3 hour then spotted onto 7H10 medium. No growth defects were observed after H_2_O_2_ treatment relative to the wild type strain mc^2^155 (Fig. 5E). In addition, no differences in growth were observed between the wild type strain mc^2^155 and the *msmeg_3312* overexpression strain after H_2_O_2_ treatment (Fig. 5E). Increased susceptibility to H_2_O_2_ was detected only when *msmeg_3312* was overexpressed in mc^2^155:Δ*2415* (Fig. 5E). In contrast, we did not detect any growth difference between the wild type strain mc^2^155, the *msmeg_6212* knockout strain mc^2^155:Δ*6212* and the *msmeg_6212* overexpression strain pMV261-*6212*/mc^2^155 after H_2_O_2_ treatment. Moreover, overexpression MSMEG_6212 in mc^2^155:Δ*2415* and the mc^2^155:Δ*2415-3312* double-knockout strain did not influence H_2_O_2_ resistance relative to the corresponding parental strains (Fig. S2A). As MSMEG_2415 is involved in the SigF-mediated H_2_O_2_ response^13^, we also measured changes in the mRNA level of *sigF* and SigF regulon components *msmeg_1782* (encoding oxidoreducatse) and *msmeg_4753* (encoding antioxidant) in mc^2^155:Δ*6212* and mc^2^155:Δ*3312* relative to that in mc^2^155. Consistent with results for H_2_O_2_ survival assays, there were no significant changes in the levels of *sigF, msmeg_1782* and *msmeg_4753* mRNA (Fig. S2B). Taken together, our results show that MSMEG_3312 has no effect on the H_2_O_2_ response in the presence of MSMEG_2415, and that MSMEG_3312 contributes to the mild H_2_O_2_ susceptibility in mc^2^155:Δ*2415*. MSMEG_6212 is not involved in the H_2_O_2_ response.

### MSMEG_2415 is not associated with erythromycin susceptibility

To further examine the role of MSMEG_2415 in erythromycin susceptibility, we overexpressed *msmeg_2415* in the mc^2^155:Δ*3312-6212* strain and examined its susceptibility to erythromycin. We incubated 15.6 mg/L erythromycin with the *msmeg_3312* knockout strain harboring a pMV261 empty vector (pMV261/ mc^2^155:Δ*3312-6212*) or the *msmeg_3312* knockout strain harboring pMV261-*2415* (pMV261-*2415*/ mc^2^155:Δ*3312-6212*) for 3h and then spotted the cells on 7H10 media. We did not detect any difference in growth between the pMV261/ mc^2^155:Δ*3312-6212* strain and the pMV261-*2415*/ mc^2^155:Δ*3312-6212* strain under drug treatment (Fig. S3A). Moreover, no differences in growth were observed between the *msmeg_3312* knockout strain (mc^2^155:Δ*3312*) and the *msmeg_3312* and *msmeg_2415* double-knockout strain (mc^2^155:Δ*3312-2415*) when treated with erythromycin (Fig. S3A). We also measured the level of *mtrA* mRNA in mc^2^155:Δ*2415*, relative to that in mc^2^155. Unlike MSMEG_3312 and MSMEG_6212, knockout of *msmeg_2415* did not affect the level of *mtrA* mRNA (Fig. S3B) and no change in the mRNA levels of MtrA regulon genes *msmeg_1875* and *msmeg_0637* was detected. Taken together, these results suggest that MSMEG_2415 is not associated with erythromycin susceptibility.

### The triple hemerythrin-like gene knockout strain mc^2^155:Δ*3312-6212-2415* exhibits comparable erythromycin susceptibility to that of mc^2^155:Δ*3312* and comparable H_2_O_2_ susceptibility to that of mc^2^155:Δ*2415*

We next constructed a triple hemerythrin-like proteins knockout strain, mc^2^155:Δ*3312-6212-2415* and confirmed it by PCR (Fig. S4). We also compared erythromycin susceptibility and H_2_O_2_ susceptibility in mc^2^155:Δ*3312-6212-2415* with that in wild type mc^2^155, mc^2^155:Δ*3312-6212,* and mc^2^155:Δ*2415*. The erythromycin susceptibility of the triple mutant strain mc^2^155:Δ*3312-6212-2415* appeared to be indistinguishable from that of the double mutant strain (mc^2^155:Δ*3312*-*6212*) (Fig. 6A). The level of *mtrA* mRNA and that of its regulon (*msmeg_1854* and *msmeg_0637*) in the triple mutant were comparable to that in mc^2^155:Δ*3312* (Fig. 6B). As expected, the H_2_O_2_ susceptibility of mc^2^155:Δ*3312-6212-2415* was comparable to that of mutant mc^2^155:Δ*2415* (Fig. 6C). The level of *sigF* and SigF regulon (*msmeg_4753* and *msmeg_1782*) mRNA in Δ*3312-6212-2415* was comparable to that in mc^2^155:Δ*2415* (Fig. 6D).

### Phylogenetic analysis of the hemerythrin-like proteins of *M. smegmatis* reveals multiple origins and distinct physiological adaptations

**Figure 7:**
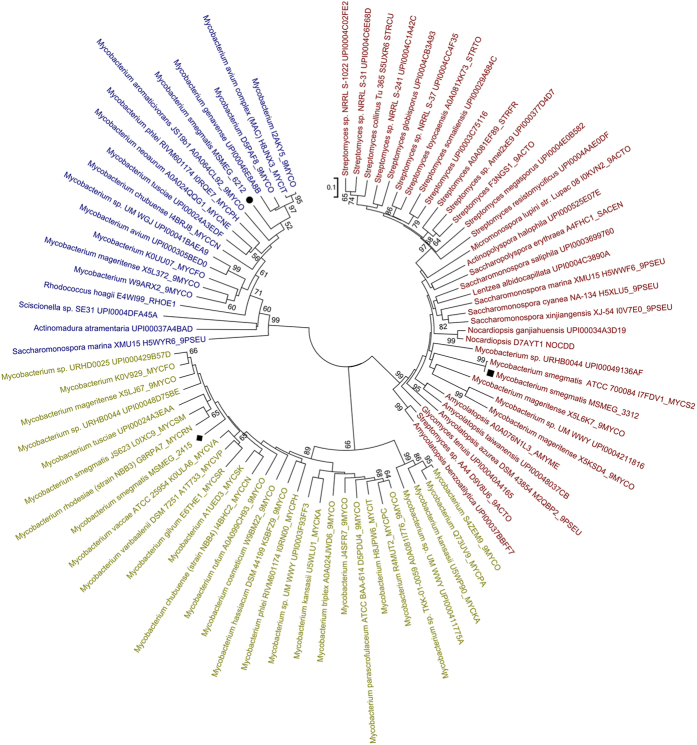
Phylogenetic relationships of mycobacterial hemerythrin-like proteins in bacteria. The unrooted neighbor-joining tree (NJ) was constructed using MEGA 5.1 software, with 1000-replicate bootstrapping. The bootstrap values less than 50% are not shown. The hemerythrin-like protein sequences used were obtained from the UniRef 90 dataset of UniProt. The scale bar represents 10% sequence divergence. Squares indicate MSMEG_3312, diamonds indicate MSMEG_2415 and circles indicate MSMEG_6212.

The above results demonstrated that the three hemerythrin-like proteins have different roles: MSMEG_3312 is associated with both erythromycin and H_2_O_2_ susceptibility, while MSMEG_2415 has a major effect on H_2_O_2_ susceptibility, and MSMEG_6212 has a role in erythromycin susceptibility. Phylogenetic analysis can help to explain the protein evolution of physiological adaptations^3^. We thus used phylogenetic analysis to gain new insights into the evolution of the *M. smegmatis* hemerythrin-like proteins. We first retrieved the sequences of the closest neighbors (top 100BLAST hits) of *M. smegmatis* MSMEG_6212, MSMEG_2415 and MSMEG_3312 from the UniRef 90 dataset of UniProt^18^, removing any duplicate sequences and then constructed a boots strapped (1000 replicate) phylogenetic relationships among the resulting 84 downloaded sequences to obtain a unrooted neighbor-joining tree (NJ) using MEGA 5.1. The used protein sequences are listed in supplemental Table S2. Interestingly, MSMEG_3312, MSMEG_2415 and MSMEG_6212 are present in 3 different clades (Fig. 7). All the proteins in the MSMEG_2415 cluster belong to the genus *Mycobacterium*, while those in the MSMEG_3312 cluster were derived from *Mycobacterium, Rhodococcus* and *Sciscionellas*. A large portion of the hemerythrin-like proteins belonging to the *Streptomyces, Saccharomonospora* and *Amycolatopsis* were present in the MSMEG_6212 cluster, suggesting that *msmeg_6212* may have an independent origin. Taken together, this phylogenetic data suggests that the origins and evolution of MSMEG_3312, MSMEG_2415 and MSMEG_6212 are different. Differences in origins may explain the differences in their physiological functions.

## Discussion

In this study, we have systematically evaluated the roles of multiple hemerythrin-like proteins (MSMEG_3312, MSMEG_2415 and MSMEG_6212) on erythromycin and H_2_O_2_ susceptibility in *M. smegmatis*. This study is the first to analyze the function and relationship between multiple hemerythrin-like proteins within one organism.

We showed that MSMEG_6212 is associated with erythromycin susceptibility but not susceptibility to the other drugs tested, including isoniazid (INH), ciprofloxacin (CIP) and rifampin (RFP) (Table S1). Erythromycin is a macrolide, a class of molecules which targets the 50S ribosome and inhibits bacterial protein synthesis^19^. WhiB7, a transcription factor for the Fe-S cluster, has been shown to be involved in inherent resistance to erythromycin, but not INH^20^. The MtrA-MtrB system has been confirmed as an essential two-component system in mycobacteria^21^,^22^. Several previous studies have shown that MtrA modulates *M. tuberculosis* proliferation by binding to the *dnaA* promoter^23^,^24^. MrtA has also been shown to be related to antimicrobial resistance in mycobacteria^15^,^16^,^17^,^25^. MtrA has been predicted to target 264 genes, including ABC transporters, ribosomal proteins, and a methyltransferase, all of which are related to drug resistance^17^. MSMEG_3312 and MSMEG_6212 are required for MtrA-mediated erythromycin susceptibility, but not for WhiB7-mediated erythromycin resistance (Fig. 4). Our results are the first to show that MSMEG_3312 and MSMEG_6212 impact the mRNA level of MtrA and thus affect its regulon, causing drug resistance. It will be interesting to explore the relationship between the WhiB7-mediated and MSMEG_6212-involved-MtrA-mediated pathways in erythromycin susceptibility.

**Figure 8:**
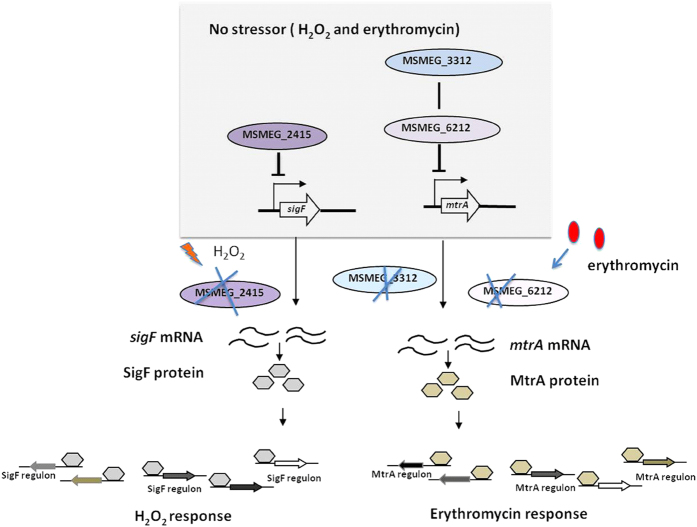
Proposed model for showing the role of *M. smegmatis* hemerythrin-like proteins in H_2_O_2_ and erythromycin susceptibility. MSMEG_3312 and MSMEG_6212 are in the same pathway and impact the mRNA level of *mtrA*, thus affecting its regulon and causing erythromycin susceptibility. MSMEG_2415 impacts the mRNA level of *sigF* and thus affects its regulon, causing H_2_O_2_ susceptibility.

Multiple homologs are common in mycobacteria. For example, *M. tuberculosis* contains five resuscitation-promoting factor (Rpf)-like proteins and ten universal stress proteins (USPs)^26^,^27^,^28^. It was not possible to determine the exact function of USP proteins Rv1996, Rv2005c, Rv2026c and Rv2028c by knockout of individual *usp* genes, suggesting that USP proteins in *M. tuberculosis* have redundant functions^26^. Similarly, reports on the *M. tuberculosis rpf* genes indicate that they have redundant roles^27^. *M. smegmatis* possesses three hemerythrin-like proteins, MSMEG_3312, MSMEG_2415 and MSMEG_6212^29^. We defined the hierarchy of biological functions among hemerythrin-like proteins (Fig. 8). The double-knockout mutant strain mc^2^155:Δ*3312-6212* showed comparable erythromycin resistance to that of the single-knockout mutant mc^2^155:Δ*3312* (Figs 5A and 6A). In addition, *msmeg_3312* influenced the level of *msmeg_6212* mRNA, but not *vice versa* (Fig 5C,D). We thus reasoned that MSMEG_3312 and MSMEG_6212 are in same pathway for erythromycin susceptibility mediated by MtrA. Knockout of the *msmeg_3312* gene leads to a decrease in the level of *msmeg_6212* mRNA and upregulation of MtrA expression (Fig. 4A). Similarly, knockout of *msmeg_6212* disrupted the regulation of MtrA (Fig. 4D). MSMEG_2415 has previously been shown to be important for H_2_O_2_ susceptibility^13^. The contribution of MSMEG_3312 was minor; the overexpression of MSMEG_3312 only slightly increased H_2_O_2_ susceptibility in mc^2^155:Δ*2415* (Fig. 5E). Taken together, these results indicate that MSMEG_2415 and MSMEG_6212 are exclusively associated with H_2_O_2_ susceptibility and erythromycin susceptibility, respectively, while MSMEG_3312 is associated with both H_2_O_2_ and erythromycin susceptibility (Fig. 8).

Phylogenetic analysis of respiratory hemerythrin-like proteins and hemocyanins shows that the distribution of analyzed respiratory proteins may partially explain physiological adaptions^3^. Comparative genomic analysis of *M. indicus prannii* suggested that multiple hemerythrin-like protein coding sequences might have been acquired by lateral gene transfer and these proteins help mycobacteria survival in different oxygen concentrations of the environment^30^. In this study, we showed that the three mycobacterial hemerythrin-like proteins have different functions and that phylogenetic analysis of hemerythrin-like proteins in *M. smegmatis* indicates that the three proteins are distributed within three distinct clades (Fig. 7). Interestingly, all the proteins in the MSMEG_2415 cluster belong to the genus *Mycobacterium*, suggesting that MSMEG_2415-like hemerythrin proteins are more conserved in mycobacteria. The role of MSMEG_2415 in H_2_O_2_ susceptibility might be an inherent function in *Mycobacterium*. In contrast, some of the hemerythrin-like proteins in the MSMEG_6212 cluster belong to the genus *Mycobacterium,* and several belong to *Saccharomonospora* and *Amycolatopsis*, suggesting that *msmeg_6212* might be of independent origin. Strikingly, the MSMEG_3312 cluster included representatives of the genera *Streptomyces, Saccharomonospora, Saccharopolyspora, Nocardiopsis,* and *Amycolatopsis*and a few *Mycobacterium* species. Of interest, *Saccharopolyspora erythraea*, an environmental soil actinomycete, can produce the natural antimicrobial agent erythromycin^31^. Soil is a highly complex and competitive environment, in which interactions between diverse organisms occur. Evolutionary pressures in soil are high due to competition for resources have significant selective advantages over competitors. *M. smegmatis* is also an environmental soil strain, the acquisition of antimicrobial-related regulatory proteins (MSMEG_3312 and MSMEG_6212) might have a selective advantage facilitating its survival. This independent phylogenetic clade may explain the different roles of MSMEG_2415 (in H_2_O_2_ susceptibility), MSMEG_6212 (in erythromycin susceptibility) and MSMEG_3312 (in both erythromycin and H_2_O_2_ susceptibility). Further work to identify the functions of *M. tuberculosis* hemerythrin-like proteins and a comparison of their hemerythrin-like protein with those in *M. smegmatis* would provide insight into the evolution and selection of virulence and antibiotic susceptibility.

In summary, we have systematically analyzed all three hemerythrin-like proteins in *M. smegmatis* and our results indicated that the three members of this protein family possess overlapping and distinct functions: MSMEG_2415 plays an important role in H_2_O_2_ susceptibility, and MSMEG_3312 and MSMEG_6212 can modulate erythromycin resistance. Phylogenetic analysis indicated that these three proteins have different evolutionary origins, possibly explaining their different physiological functions. The functional and phylogenetic analyses of hemerythrin-like proteins in *M. smegmatis* would provide insight into the evolutionary selection of antimicrobial resistant traits.

## Materials and Methods

### Reagents and Media

As previously described^13^, the 7H9 liquid culture medium for *M. smegmatis* strains consisted of Middlebrook 7H9 medium (Becton Dickinson, Franklin Lakes, New Jersey, U.S.) supplemented with 10% ADS (5% (w/v) bovine serum albumin fraction V, 2% (w/v) dextrose and 8.1% (w/v) NaCl), 0.5% (v/v) glycerol, and 0.05% (v/v) Tween80. 7H10 media containing Middlebrook 7H10 medium (Becton Dickinson, Franklin Lakes, New Jersey, U.S.), 10% ADS, and 0.5% (v/v) glycerol was used for solid culture to examine growth status. Hygromycin (Hyg) was purchased from GenView, erythromycin (EM) from Merck, hydrogen peroxide (H_2_O_2_) and kanamycin (Kan) from Sigma. Restriction enzymes such as Van91I, AlwNI, MfeI, and PacI were purchased from Fermentas. T4 DNA ligase and Q5 DNA polymerase were purchased from New England Biolabs.

### Construction of knockout hemerythrin-like protein MSMEG_6212 strain and corresponding complemented and overexpression strains

Mycobacteriophage-based specialized transduction was used to generate hemerythrin-like gene knockout strains^13^,^32^. Briefly, the 5’ and 3’ sequences flanking the *msmeg_6212* gene were amplified from *M. smegmatis* genomic DNA using the following PCR conditions: 98 °C for 3 min, 32 cycles of 98 °C for 30 s, 60 °C for 30 s, and 72 °C for 30 s, and 72 °C for 10 min. The primers for *msmeg_6212* knockout are listed in Supplemental Table S3, and the corresponding positions are indicated in Fig. 2A. The primers used had a PflMI site on the 5’ end to allow insertion into the pYUB1471 vector, and the temperature sensitive phage phAE159 and pYUB1471-*6212* were then digested with PacI and ligated using T4 DNA ligase to create a shuttle plasmid. MaxPlax packaging extract (Epicenter Biotechnologies, USA) was used for phage packaging and transformed into *E. coli* HB101 cells according to the manufacturer’s instructions. Successful phasmids were transduced into *M. smegmatis* strain mc^2^155 at 30 °C, which allowed replication and amplified high titer phages. Transduction into *M. smegmatis* was then performed at 37 °C with the high titer lysate at an MOI of 10:1. Gene knockout was confirmed by PCR screening using primers outside the upstream and downstream flanking regions and the corresponding vector primers. Complemented strain of *msmeg_6212* was constructed by cloning the full-length genes into the integrating vector pMV361 to yield pMV361-*6212/* mc^2^155:Δ*6212.*

To obtain the *msmeg_3312* and *msmeg_6212* double knockout strain, we unmarked the mc^2^155:Δ*3312* strain used in previous study^12^ according to a previously published method^33^. Briefly, plasmid pJH532 was transformed into the mc^2^155:Δ*3312* strain by electroporation and plated onto 7H10 media containing 25 mg/L kanamycin. Kanamycin resistant colonies were screened by a pick-and-patch method for hygromycin sensitivity, streaked on 7H10 media alone and on 7H10 media with 50 mg/L hygromycin. The hygromycin-sensitive colonies were then plated onto 7H10 media with 5% sucrose. The selected colonies were spread on 7H10 media supplemented with 5% sucrose to obtain kan^S^hyg^S^ colonies. The unmarked mc^2^155:Δ*3312* strain was used for construction of the *msmeg_6212* knockout strain, yielding the mc^2^155:Δ*3312*-*6212* double-knockout strain. The triple mutant mc^2^155:Δ*3312*-*6212-2415* was generated from the double-knockout progenitor mc^2^155:Δ*3312*-*6212*.

To overexpress hemerythrin-like proteins, the corresponding full-length coding genes, *msmeg_3312, msmeg_2415* and *msmeg_6212*, were sub-cloned into pMV261 to yield pMV261-*3312*, pMV261-*2415* and pMV261-*6212* for transformation into the corresponding *M. smegmatis* strains (All strains used in this study are listed in Table S4).

### Drug and H_2_O_2_ susceptibility testing

The killing curve under erythromycin treatment was determined as indicated. Logarithmic phase cultures (OD_600_ ~ 0.3) were treated with erythromycin at the indicated concentrations, aliquots (~50 μl) were removed at the indicated times and spread onto 7H10 medium. Colony Forming Units (CFUs) were counted after 3 days of incubation. Experiments were repeated at least 3 times.

Survival under erythromycin and H_2_O_2_ treatment was determined as indicated. Logarithmic phase cultures (OD_600_ ~ 0.3) were treated with erythromycin (15.6 mg/L) or H_2_O_2_ (5 mM) at the indicated concentrations for 3 h, serially diluted (1:10) and spotted (3 μl) onto 7H10 medium. Photographs were taken after three days of incubation at 37 °C. Experiments were repeated at least 3 times.

### Quantitative real-time PCR analysis

Logarithmic phase (OD_600_ ~ 0.3)cultures of the corresponding *M. smegmatis* strains treated with 0 or 3.125 mg/L erythromycin for 30 min were collected. After resuspending in TRIzol (Invitrogen), RNA was purified according to the manufacturer’s instructions. The SuperScriptTM III First-Strand Synthesis System (Invitrogen) was used to synthesize the corresponding cDNA. qRT-PCR was performed on a Bio-Rad iCycler. *M. smegmatis rpoD* (the coding sequencing of RNA polymerase sigma factor SigA) was used to normalize gene expression. The relative ratio was calculated using the 2^−ΔΔCT^ method^34^. Experiments were repeated at least 3 times. Primers used for qRT-PCR are listed in Table S3.

### Statistical method

Statistical analysis was performed with GraphPad Prism 5.0c software. Significant differences in the data were determined using t-tests. P values of <0.05 were considered significant.

## Additional Information

**How to cite this article**: Li, X. *et al.* Differential roles of the hemerythrin-like proteins of *Mycobacterium smegmatis* in hydrogen peroxide and erythromycin susceptibility. *Sci. Rep.* **5**, 16130; doi: 10.1038/srep16130 (2015).

## Supplementary Material

Supplementary Information

Supplementary Information
