# Supplementary material for: Differential roles of the hemerythrin-like proteins of Mycobacterium smegmatis in hydrogen peroxide and erythromycin susceptibility
Source: Sci Rep. 2015 Nov 26;5:16130. doi: 10.1038/srep16130 (PMC4660385; doi:10.1038/srep16130)
Supplement: Supplementary Information [file srep16130-s1.doc]

**Differential roles of the hemerythrin-like proteins of *Mycobacterium smegmatis* in hydrogen peroxide and erythromycin susceptibility**

Xiaojing Li^1#^, Jingjing Li^1, 2#^, Xinling Hu^1^, Lige Huang^1^, Jing Xiao^1^, John Chan^3^, Kaixia Mi^1, 4*^

^1^ CAS Key Laboratory of Pathogenic Microbiology and Immunology, Institute of Microbiology, CAS, Beijing 100101, China

^2^ School of Life Sciences, Anhui University, Hefei 230601, Anhui Province, China

^3^ Division of Infectious and Diseases, Department of Medicine, Albert Einstein College of Medicine, New York 10461, USA

^4^ Beijing Key Laboratory of Microbial Drug Resistance and Resistome, Beijing 100101, China

**Supplementary Figures:**

**
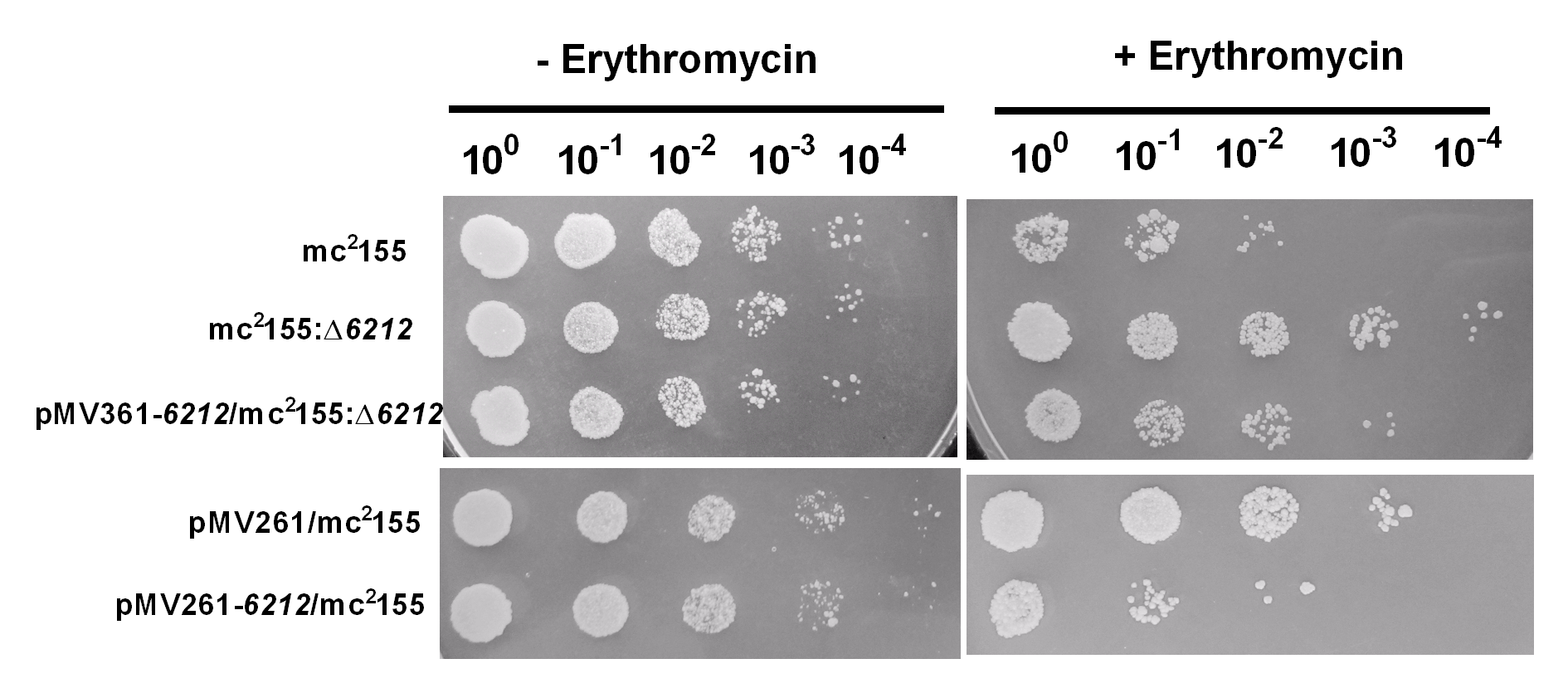
**

**Supplementary Figure 1. MSMEG_6212 is involved in erythromycin susceptibility.** Serial dilutions (1:10) of mc^2^155, mc^2^155:Δ*6212* and the complementary strain pMV361-6212/mc^2^155:Δ*6212*, overexpression strain pMV261-*6212*/mc^2^155 growth on 7H10 media and the survival of corresponding strains spotted on 7H10 media after 3 hour of treatment with 15.6 mg/L erythromycin．Photographs were taken after three days of incubation at 37°C. The pictures shown are representative of three independent experiments．


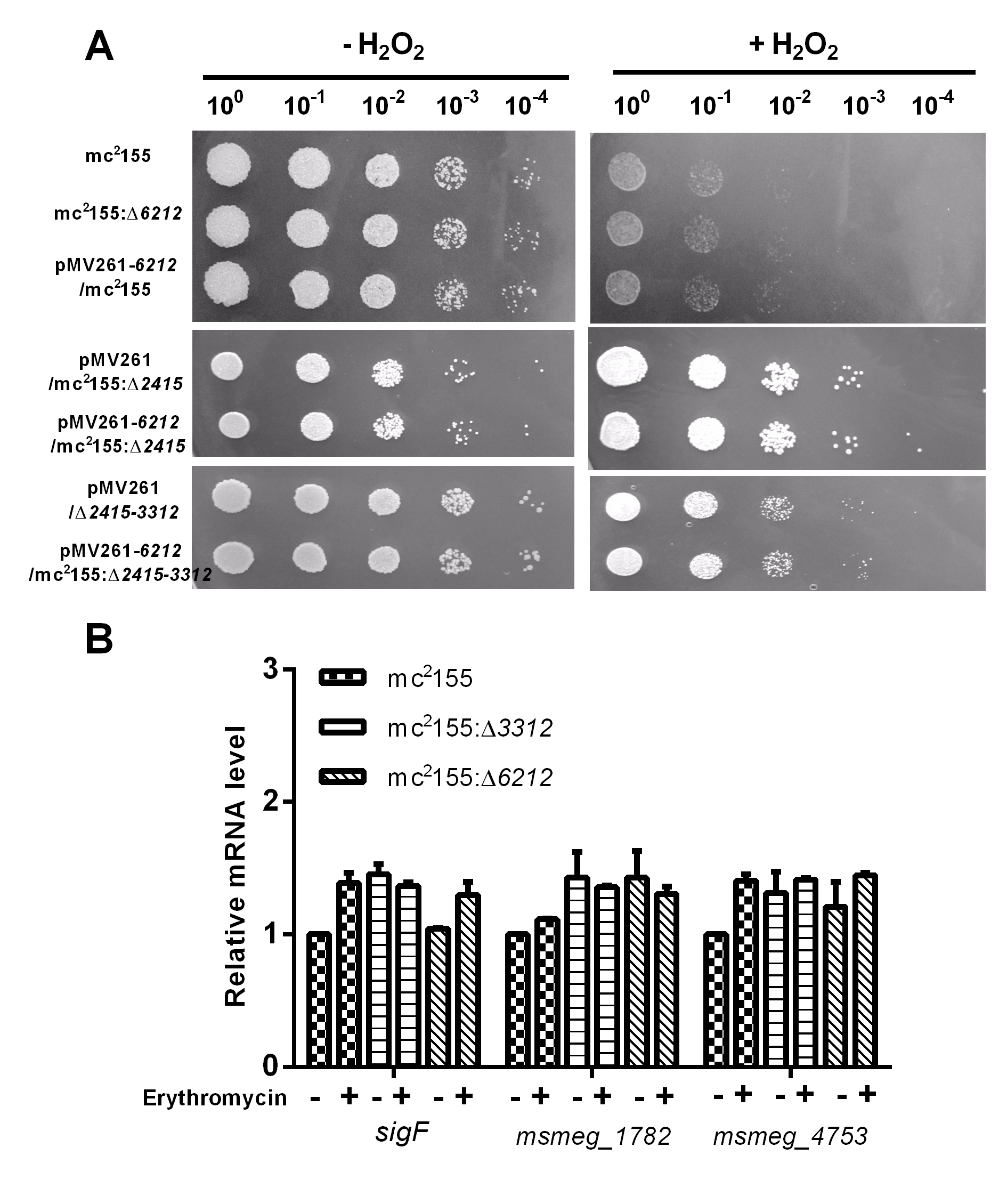


**Supplementary Figure 2. MSMEG_6212 is not involved in H_2_O_2_ susceptibility.**  **A.** The growth of serial dilutions (1:10) of mc^2^155, Δ*6212*, the overexpression strain pMV261-*6212*/mc^2^155, pMV261-*6212*/ mc^2^155:Δ*2415*, pMV261-*6212*/ mc^2^155:Δ*2415-3312* on 7H10 media and the survival of corresponding strains spotted on 7H10 media after 3 hour of treatment with 5 mM H_2_O_2_．Photographs were taken after three days of incubation at 37°C. The pictures shown are representative of three independent experiments. **B.** Relative expression levels of *sigF*, *msmeg_1782*, *msmeg_4753* in Δ*3312*, Δ*6212* and mc^2^155 without or with erythromycin treatment.

**
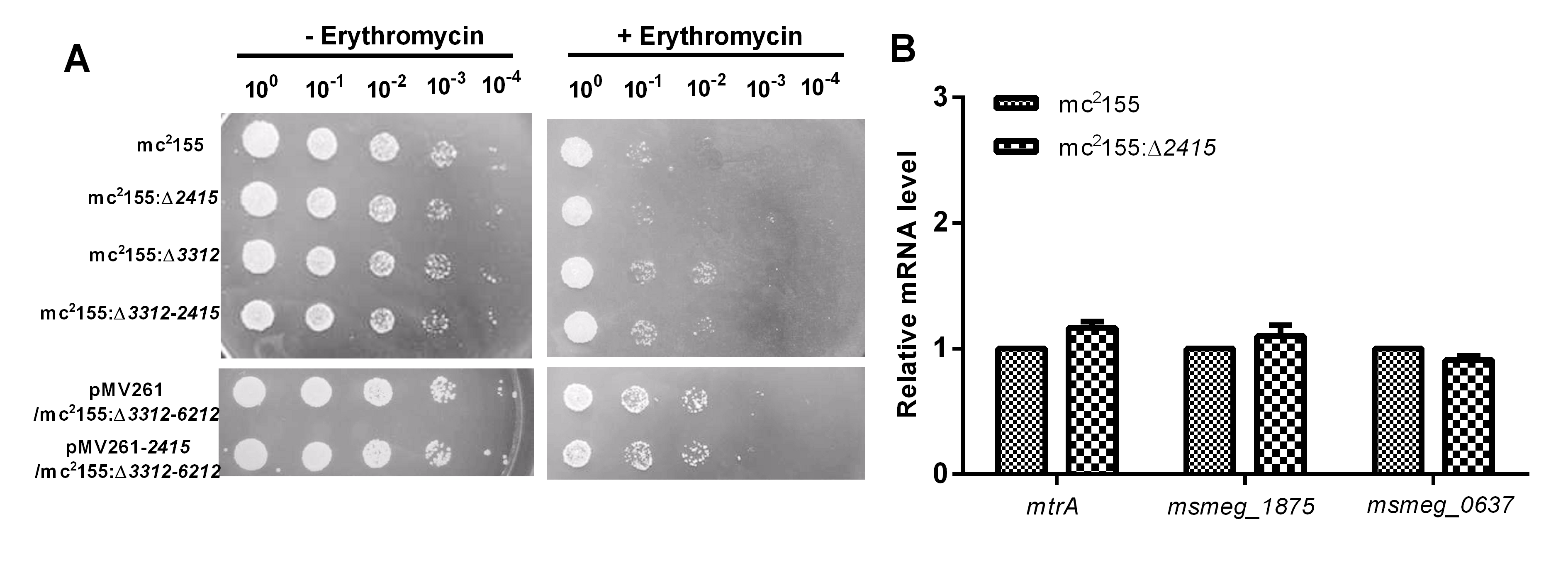
**

**Supplementary Figure 3. MSMEG_2415 is not involved in erythromycin susceptibility. A.** Effect of mc^2^155, mc^2^155:Δ*2415*, mc^2^155:Δ*3312*, mc^2^155:Δ*3312-2415* and pMV261/ mc^2^155:Δ*3312-6212*, pMV261-*2415*/ mc^2^155:Δ*3312-6212* on erythromycin susceptibility. Cells from 0.3 of OD_600_ were incubated for 3 hour with 15.6 mg/L erythromycin. The growth of serial dilutions (1:10) of the indicated strains on 7H10 media and the survival of corresponding strains spotted on 7H10 media. Photographs were taken after three days of incubation at 37°C. The pictures shown are representative of three independent experiments. **B.** Relative expression levels of *mtrA*, *msmeg_1874*, *msmeg_0637* in mc^2^155:Δ*2415* and mc^2^155.


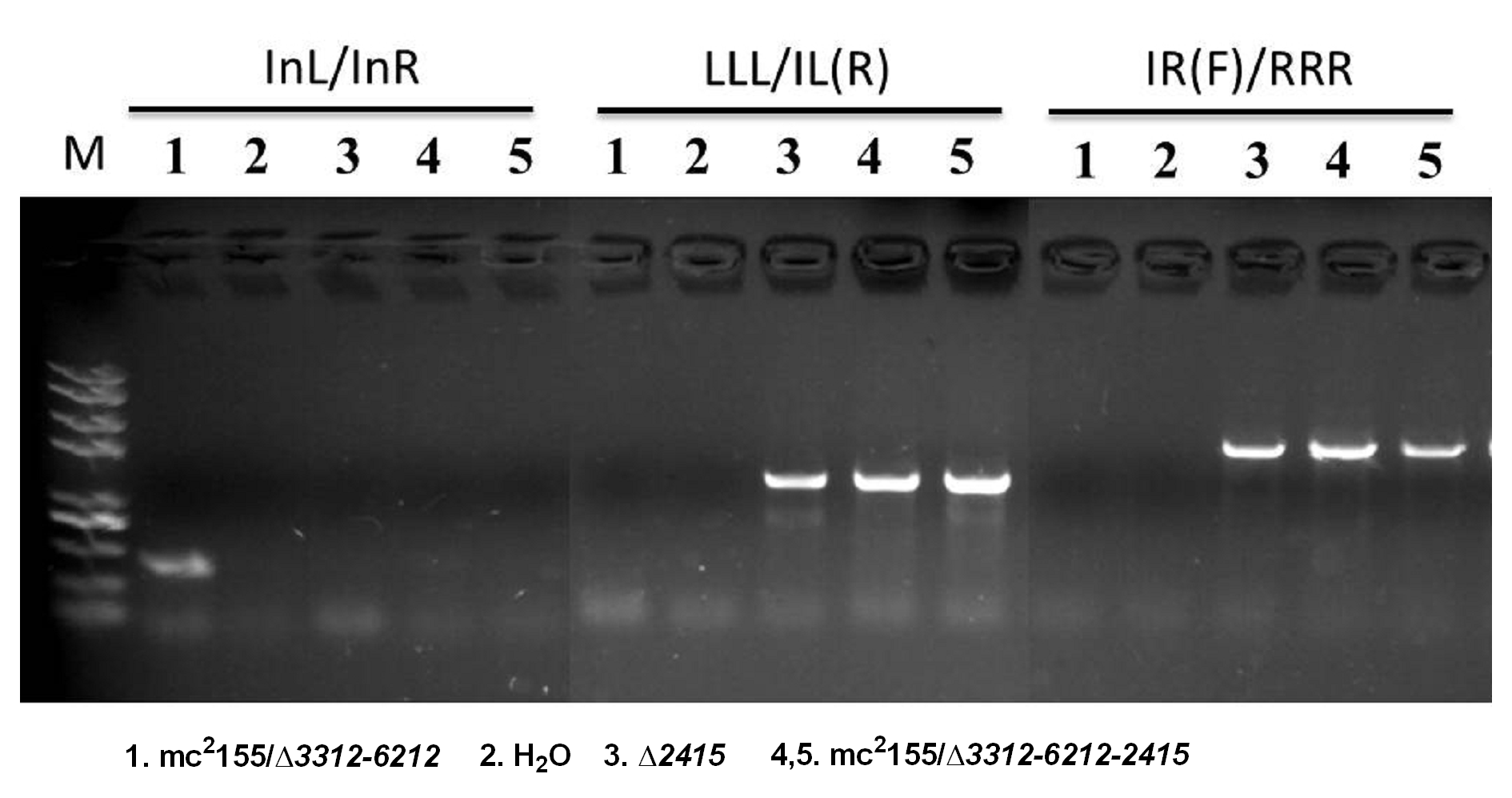


**Supplementary Figure 4.** **Identification of *msmeg_2415* knockout in mc^2^155:**△***3312-6212*.** The *msmeg_2415* knockout was confirmed by PCR screening using *msmeg_2415* interior primer (2415InL/2415InR) and primers outside the upstream and downstream flanking regions and the corresponding vector primers (2415LLL/IL(R) and IR(F)/2415RRR). The numbers 1,3 represent the genomic DNA of mc^2^155:Δ*3312-6212*, mc^2^155:Δ*2415* as template; 4, 5 represent the genomic DNA of mc^2^155:Δ*3312-6212-2415* as template; 2 represents H_2_O as template, which is the negative control for PCR.

**Supplementary Tables:**

***Supplementary Table 1: Minimum inhibitory concentrations (MICs) of tested drugs and H_2_O_2_ in mc^2^155, mc^2^155:Δ2415, mc^2^155:Δ3312, and mc^2^155: Δ6212***

| MIC* | INH* | RFP | EMB | EM | AZM | CM | GM | H_2_O_2_ | TET | OT | STR | CIP |
| --- | --- | --- | --- | --- | --- | --- | --- | --- | --- | --- | --- | --- |
| **mc^2^155** | 25 | 3.125 | 1.56 | 3.125 | 1.56 | 12.5 | 1.56 | 0.039 | 0.195 | 0.195 | 0.39 | 0.156 |
| **mc^2^155:△*2415*** | 25 | 3.125 | 1.56 | 3.125 | 1.56 | 12.5 | 1.56 | 0.039 | 0.195 | 0.195 | 0.39 | 0.156 |
| **mc^2^155:△*3312*** | 25 | 3.125 | 1.56 | 12.5 | 3.125 | 12.5 | 1.56 | 0.039 | 0.195 | 0.195 | 0.39 | 0.156 |
| **mc^2^155:△*6212*** | 25 | 3.125 | 1.56 | 6.25 | 3.125 | 12.5 | 1.56 | 0.039 | 0.195 | 0.195 | 0.39 | 0.156 |

*mg/L，except H_2_O_2_ (mM)

*INH: Isoniazid, RFP: rifampicin, EMB: ethambutol, EM: erythromycin, AZM: azithromycin,

CM: chloramphenicol, GM: gentamicin, TET: tetracycline, OT: oxytetracycline, STR: streptomycin, CIP: ciprofloxacin

**Supplementary Table 2. The used protein sequences for phylogenetic analysis**

supplementary dataset as separate Excel document.

**Supplementary Table 3**. Oligonucleotide primers used in this study*

| Name | 5’-3’ | purpose |
| --- | --- | --- |
| 6212LL | TTTTTTTTCCATAAATTGGACGTCACCTTCGCGAGCCT | *msmeg_6212*  gene knockout |
| 6212LR | TTTTTTTTCCATTTCTTGGGATTCGGCGATGATCAGGTT |
| 6212RL | TTTTTTTTCCATAGATTGGACCGGCTGCTGATTCTTGTC | *msmeg_6212*  gene knockout |
| 6212RR | TTTTTTTTCCATCTTTTGGAGAACCCGAAGTTGTACGAC |
| 3312LF | TTTTTTTTCCATAAATTGGTGTCGGCGCTTACCACATCT | *msmeg_3312*  gene knockout |
| 3312LR | TTTTTTTTCCATTTCTTGGTTCGACCAGTTCCGGTTGTG |
| 3312RF | TTTTTTTTCCATAGATTGGTGAGTGAAGAGTTTCAGCGT | *msmeg_3312*  gene knockout |
| 3312RR | TTTTTTTTCCATCTTTTGGAAAGTCCAAGCAGGTCAGCG |
| 2415LL | TTTTTTTTCCATAAATTGGTTCAACAGCGACCTCGTGAC | *msmeg_2415*  gene knockout |
| 2415LR | TTTTTTTTCCATTTCTTGGTGTGCTGATCGGTGAGAAAC |
| 2415RL | TTTTTTTTCCATAGATTGGATCGGCTCGGCCCTGAA | *msmeg_2415*  gene knockout |
| 2415RR | TTTTTTTTCCATCTTTTGGTTCGCATGCGTCGCCATA |
| 6212LLL | ACACTGATCGGCAACCGTTTCG | *msmeg_6212*  knockout identification |
| 6212RRR | ATCCGCCACCAATCTGCAATCAAC |
| 6212InL | ATCGAGGAGCAGCACTTCTGG | *msmeg_6212*  knockout identification |
| 6212InR | ATGCCCATGGTCTTCTGTACGAC |
| 3312LLL | TTGTCGCGTACATCATCCTG | *msmeg_3312*  knockout identification |
| 3312RRR | TCGGCATCAGCAGCGGAA |
| 3312InL | CGTGATTTCTGGCATCGTG | *msmeg_3312*  knockout identification |
| 3312InR | ACTCGTGCAGCCGCTCG |
| 2415LLL | TACGACGTCGACGCACGCAC | *msmeg_2415*  knockout identification |
| 2415RRR | CGATGAGATCCAAGCCGATTCGT |
| 2415InL | AACCTGATCAAGGACCTCTTCG | *msmeg_2415*  knockout identification |
| 2415InR | CGACGGCGAAGTTCAGCGA |
| IL(R) | TCGACGACCCTAGAGTCC | knockout identification |
| IR(F) | GACACACCAACAGCATGGT |
| Msm6212pMVF | CCGGAATTCATGGATGGTTTGACATTTCTG | pMV261- *6212*  pMV361- *6212* |
| Msm6212pMVR | CCCAAGCTTTCAGTGGATCTGGGGGTC |
| Msm3312pMVF | CGGGAATTCATGGCTGGTAGCAGAGGCAA | pMV261- *3312* |
| Msm3312pMVR | CGGAAGCTTTCAGCTGTTGCGGCCGCT |
| Msm2415pMVF | AGCGGAATTCGTGGCCGATTCAAAGCCCA | pMV261- *2415* |
| Msm2415pMVR | ACGTGGATCCCACGTCAGCGCTACTTCAG |
| MsmRpoDqF | GTGTGGGACGAGGAAGAGTC | qRT-PCR |
| MsmRpoDqR | ACCTCTTCTTCGGCGTTGAG |
| Msm6212qF | AGCACTTCTGGCCGCTCGTG | qRT-PCR |
| Msm6212qR | GCTTGCCGTCTTCGAGGCGT |
| Msm3312qF | CGAGCGGCTGCACGAGTTGA | qRT-PCR |
| Msm3312qR | TTGTTGGCCGGCGGATGGTC |
| Msm1804qF | CGGCTCAAGGAACTCCACTT | qRT-PCR |
| Msm1804qR | TCTTCGCGATCCATGTCCAG |  |
| Msm1782qF | TGGCGGCACACATTCGCAGT | qRT-PCR |
| Msm1782qR | TTGCCGCGCAGTCCGTTGAT |  |
| Msm4753qF | CCGGGGCCGAAAGGTCATCG | qRT-PCR |
| Msm4753qR | CTCGGGCTTGTCGGGGGAGA |  |
| Msm1874qF | ATGAAGCCGTTCAAGCCGAA | qRT-PCR |
| Msm1874qR | TCACCGATCGACAGCATCTC |  |
| Msm1875qF | GCAGCTCGAAGAGTTCGGTA | qRT-PCR |
| Msm1875qR | TCCTCGCTGTGGTCGTAGAT |  |
| Msm0637qF | AGGACTCGTCGGGACTGAAG | qRT-PCR |
| Msm0637qR | CGAGAATCGGTATCCCCGTG |  |

* Restriction enzyme sites are underlined.

**Supplementary** Table 4. Bacterial strains and plasmids used in this study

| Name | Relevant genotype or description | Reference |
| --- | --- | --- |
| **Strains** |  |  |
| *Mycobacterium smegmatis* |  |  |
| mc^2^155 | Wild type | W.R. Jacobs |
| mc^2^155:Δ*6212* | Deletion mutant of *msmeg_6212* | This work |
| mc^2^155:Δ*3312* | Deletion mutant of *msmeg_3312* | This work |
| mc^2^155:Δ*2415* | Deletion mutant of *msmeg_2415* | This work |
| mc^2^155:pMV361-*6212*/Δ*6212* | Δ*6212* containing pMV361-*6212* which contains full length *msmeg_6212* |  |
| pMV261-*6212*/mc^2^155 | mc^2^155 containing pMV261-*6212* which contains full length *msmeg_6212* | This work |
| pMV261-*3312*/mc^2^155 | mc^2^155 containing pMV261-*3312* which contains full length *msmeg_3312* | This work |
| mc^2^155:Δ*3312-6212* | Deletion mutant of *MSMEG_3312* and *msmeg_6212* | This work |
| mc^2^155:Δ*3312-2415* | Deletion mutant of *MSMEG_3312* and *msmeg_2415* | This work |
| mc^2^155:Δ*3312-2415-6212* | Deletion mutant of *msmeg_3312*, *msmeg_2415*, *msmeg_6212* | This work |
| pMV261-*3312*/ mc^2^155:Δ*2415* | Δ*2415* containing pMV261-*3312* which contains full length *msmeg_3312* | This work |
| pMV261-*2415*/ mc^2^155:Δ*3312* | Δ*3312* containing pMV261-*2415* which contains full length *msmeg_2415* | This work |
| pMV261-*2415*/ mc^2^155:Δ*3312-6212* | Δ*3312-6212* containing pMV261-*2415* which contains full length *msmeg_2415* | This work |
| pMV261-*6212*/ mc^2^155:Δ*2415* | Δ*2415* containing pMV261-*6212* which contains full length *msmeg_6212* | This work |
| pMV261-*6212*/ mc^2^155:Δ*3312-2415* | Δ*3312-2415* containing pMV261-*6212* which contains full length *msmeg_6212* | This work |
| **Plasmids** |  |  |
| pMV261 | *Mycobacterial* extrachromosomal expression vector, Km^R^ | [^1^](#_ENREF_1) |
| pMV361 | *Mycobacterial* integrative expression vector, Km^R^ | [^1^](#_ENREF_1) |
| phAE159 | Temperature-sensitive mycobacteriophage phasmid, Amp^R^ | [^2^](#_ENREF_2) |
| pYU1471 | Clone vector for construction allelic exchange substrate plasmid, Hyg^R^ | [^2^](#_ENREF_2) |

Amp^R^, Hyg^R^ and Km^R^ indicate resistance to ampicillin, hygromycin and kanamycin, respectively.

1. Stover, C.K. et al. New use of BCG for recombinant vaccines. *Nature* **351**, 456-460 (1991).

2. Bardarov, S. et al. Specialized transduction: an efficient method for generating marked and unmarked targeted gene disruptions in Mycobacterium tuberculosis, M. bovis BCG and M. smegmatis. *Microbiology* **148**, 3007-3017 (2002).
